# InCoB celebrates its tenth anniversary as first joint conference with ISCB-Asia

**DOI:** 10.1186/1471-2164-12-S3-S1

**Published:** 2011-11-30

**Authors:** Christian Schönbach, Tin Wee Tan, Janet Kelso, Burkhard Rost, Sheila Nathan, Shoba Ranganathan

**Affiliations:** 1 Department of Bioscience and Bioinformatics, Graduate School of Computer Science and Systems Engineering, Kyushu Institute of Technology, Fukuoka 820-8502, Japan; 2 Department of Biochemistry, Yong Loo Lin School of Medicine, National University of Singapore, Singapore 117597; 3 Department of Evolutionary Genetics, Max Planck Institute for Evolutionary Anthropology, 04103 Leipzig, Germany; 4 Department for Bioinformatics and Computational Biology, Faculty for Informatics, Technical University Munich, 85748 Garching, Germany; 5 School of Biosciences and Biotechnology, Faculty of Science and Technology, Universiti Kebangsaan Malaysia, 43600 UKM Bangi Selangor D.E., Malaysia; 6 Malaysia Genome Institute, Jalan Bangi, 43000 Kajang Selangor D.E., Malaysia; 7 Department of Chemistry and Biomolecular Sciences and ARC Centre of Excellence in Bioinformatics, Macquarie University, Sydney NSW 2109, Australia

## Abstract

In 2009 the International Society for Computational Biology (ISCB) started to roll out regional bioinformatics conferences in Africa, Latin America and Asia. The open and competitive bid for the first meeting in Asia (ISCB-Asia) was awarded to Asia-Pacific Bioinformatics Network (APBioNet) which has been running the International Conference on Bioinformatics (InCoB) in the Asia-Pacific region since 2002. InCoB/ISCB-Asia 2011 is held from November 30 to December 2, 2011 in Kuala Lumpur, Malaysia. Of 104 manuscripts submitted to BMC Genomics and BMC Bioinformatics conference supplements, 49 (47.1%) were accepted. The strong showing of Asia among submissions (82.7%) and acceptances (81.6%) signals the success of this tenth InCoB anniversary meeting, and bodes well for the future of ISCB-Asia.

## Introduction

The tenth InCoB (International Conference on Bioinformatics), an official conference of the Asia-Pacific Bioinformatics Network (APBioNet) [1], was held in Kuala Lumpur, Malaysia as a joint conference with the ISCB-Asia meeting of the International Society for Computational Biology (ISCB) [2]. InCoB/ISCB-Asia 2011 is the result of ISCB’s mission to host meetings in locations that are not well served by the world’s largest bioinformatics conference ISMB (International Conference on Intelligent Systems for Molecular Biology). Owing to the success of previous InCoB conferences [3,4], and as ISCB’s first regional affiliate, APBioNet was selected to co-organize the first ISCB-Asia conference.

## Manuscript submission and review

We offered authors three tracks to submit manuscripts for potential publication in the supplement issues of *BMC Bioinformatics* or *BMC Genomics* (BMC track) and *Immunome Research* (IR) [5]. Of 110 submitted manuscripts from 19 countries 104 were designated for the BMC track. Immunome Research received three submissions. Shorter manuscripts representing research in progress were also sought for Bioinformation (BI), which received three submissions. Most manuscripts received three reviews from the 76 member strong Program Committee, supported by 39 additional reviewers (Additional File 1). The first round of reviews resulted in the provisional acceptance of 32 (30.8%) manuscripts, with minor revisions. The authors of 28 manuscripts, including three that were transferred by the Program Committee Co-chairs to BI and IR tracks, had to address major concerns raised by the reviewers. After a second round of review, 26 (25%) manuscripts were provisionally accepted pending minor revisions. A statistics of submissions and final acceptances by countries is shown in Additional File 2. The manuscript revision and re-review policy of InCoB/ISCB-Asia 2011 resulted in a more than twofold greater acceptance rate compared to ISMB/ECCB 2011 [6] which considers only manuscripts requiring minor revisions.

The 25 articles selected for this issue represent a cross-section of scientific and technological innovations accelerating bioinformatics in the areas of databases, software tools, RNomics, next-generation sequencing, sequence analysis, evolution, proteome analysis and disease informatics. The remaining 24 accepted BMC articles are published as a BMC Bioinformatics supplement [7].

## Biological databases and software tools

Papers presenting a wide variety of databases and software tools were accepted [8-12]. Particular focus areas include the development of software for the alignment of apicomplexan sequences [8] which have an unusual sequence composition, software for the identification of gene regulatory modules from microarray data [9], and software for the identification of taxonomic identity from metagenomic samples [10]. Liverome [11] and Detoxiprot [12] represent databases for collecting and sharing of network modules enriched in liver cancer-pathways as well as enzymes, substrates, inducers and inhibitors of detoxification.

## RNomics and next-generation sequencing

Four papers make use of next-generation sequencing (NGS) [13-16] to address a range of challenges from the identification of the taxonomic origin of organism sequences in metagenomic studies [13] to the identification of unique and novel genes in metatranscriptomic data [14,15].

Fast-growing trees from plantations are used in pulp and fiber production. Lignin, an essential component of the tree structure, is undesired in this process. Ong and Wickneswari [16] applied NGS to profile the expression of *Acacia mangium* small RNAs in secondary xylem samples of varying lignin content. The obtained small RNA profiles, which include 82 novel miRNAs may open venues to silence monolignol biosynthesis-associated genes without compromising the fitness of the tree.

Secretory proteins are important players in host-pathogen interactions. Existing prediction tools do not support the identification of secretory protein candidates from NGS data, thus hampering the identification of non-classical secreted protein candidates. Garg and Ranganathan [17] developed an assembly protocol for NGS-derived sequence data to cluster, translate and homology search an in-house dataset of experimentally determined parasitic helminth excretory/secretory proteins. Another aspect of the cellular secretome is exosomes or vesicles used in intracellular communication and transport of cellular components such as RNA. Since exosomes could be used as vectors for therapeutic drug delivery, it is of interest to identify motifs involved in targeting RNA for secretion. Bagatov and colleagues [18] determined *ab initio* various short linear motifs that are specific for certain exosome-enriched RNAs.

## Evolution and sequence analysis

The growing number of complete genome drafts and protein sequence data allows to re-visit questions on the origin of vertebrate promoters, evolutionary conservation of enzymes, as well as the generation of functional diversity through gene duplication and alternative splicing. Profiling of ascidian promoters revealed a primordial vertebrate promoter type that appears to be partially methylated with a high but limited extent of CpG scores and G+C content [19]. Phylogenetic profiles of rate-limiting enzymes with inhibiting relations showed higher conservation across human, rat, mouse, budding yeast and *E. coli* than common enzymes [20]. Chen *et al*. [21] analysed the relationship between the two mechanisms using protein sequence and paralog information of seven organisms ranging from human to *C. elegans*. The results indicate a duplication age-dependent relationship of alternative splicing and an evolutionary constraint among duplicates. Alternative splicing was more frequently observed among ancient than recent duplicates.

The increasing scale and speed of whole-genome sequencing requires faster and high-accuracy genome mapping methods. A new method that utilizes perfect Hamming Code with a hash table significantly reduces the number of hash keys required for searching genome positions [22]. The Universal Automatic SNP Identification System (UASIS) [23], cross-references SNP identifiers of various databases to bridge potential SNP nomenclature ambiguities and to promote compliance with Human Genome Variation Society guidelines.

## Proteome analysis

Knowledge of protein-protein interaction (PPI) data quality and their limitations is critical for interpreting results and essential for using them as a reference set. Zhou and Wong [24] found that *M. tuberculosis* H37Rv bacterial two-hybrid (B2H) data seemed to contain numerous false positives and false negatives, while the H37Rv STRING predicted PPIs included many none-direct interactions. Yet, stringently predicted H37Rv STRING PPI data appeared to be suitable as a reference for analysing functional associations rather than physical interactions. To understand the behaviour of proteins in PPI networks, PPIs are often correlated with gene expression parameters. The correlation of high-confidence human PPIs derived from the HitPredict database with gene expression stability improved the classification of transient obligatory hubs and revealed functionally distinct, previously unknown type of hubs [25].

All protein domains common to mouse-infecting viruses and mice were analyzed and published as MusVirus database and tool [26]. Biochemical and functional interpretations of the shared domains indicated that the analysed viruses prefer to acquire genes of the innate immune response pathways. Granzymes are serine proteases that are implicated in the pathogenesis of several chronic inflammatory and cardiovascular disorders. Wee *et al*. [27] implemented a webserver to predict the potential granzyme B degradome using support vector machine classifiers derived from experimentally verified substrate cleavage sites.

Beta-D-mannosidase mutations cause lysosomal storage disease. A comparative structural bioinformatics analysis of inherited mutations in β-D-mannosidase across multiple species revealed a geno-phenotpye correlation [28]. The identification of five mutational hotspots indicated that the closeness of mutations to the active site correlates with the severity of phenotypes and thus allowing phenotype severity predictions.

## Disease informatics

Papers on the discovery and analysis of biomarkers and disease gene candidates focussed on copy number variations (CNVs) [29], cut-circularize-linearize-and-paste (CCLP) models [30] of chromosomal rearrangements and an extreme class-discrimination (ECD) [31] of lung adenocarcinoma gene expression data. Shia *et al*. [29] examined CNVs in Taiwanese hyperlipidemia and myocardial infarction patient cohorts. A multistage analysis revealed seven CNV regions that were associated with both disorders and deserve further exploration as potential biomarkers for early-stage diagnosis. To study the human immune response to tumours and phylogenetic tree reconstructions, Huang *et al *[30] developed an algorithm that minimizes the number of CCLP operations to sort permutations of a number of genes in a given chromosome.

Lack of clinically applied biomarkers for the early diagnosis and aggressiveness of lung adenocarcinoma inspired the development ECD to select features in paired gene expression samples, and to identify genes that are essential for reprogramming lung tissue cells [31]. Compared to commonly used methods, ECD produced highly discriminative variables when the number of samples was small. Nevertheless, prioritizing and experimentally verifying new candidates can be time-consuming and expensive. Hsu *et al*. [32] proposed a parameter-free interconnectedness method to rank disease candidates by evaluating their distance to known disease genes in a network.

## Conclusion

In recent years numerous Asian bioscience conferences have added bioinformatics sessions to their program. Yet, higher-profile annual conferences dedicated to bioinformatics remained largely in North America and Europe. The high number of manuscript and poster submissions to InCoB/ISCB-Asia 2011 suggests that the joint conference model ISCB-Asia may indeed invigorate regional bioinformatics-themed meetings in Asia, which are necessary to advance bioinformatics research, particularly in South and Southeast Asia. The strengthening of these regional conferences should lead to a gradual and healthy consolidation of smaller meetings that will benefit participants. Towards this end, we expect to see you again at the 11^th^ InCoB in Bangkok or at the 2^nd^ ISCB-Asia conference.

## Competing interests

The authors declare that they have no competing interests.

## Authors' contributions

CS and SR (Program Committee Co-chairs) wrote the introduction and managed the review and editorial processes. CS, SR, JK (Chair, ISCB Conferences Committee), BR, TWT and SN (Conference Chair) jointly contributed to the scientific program development and its implementation. TWT supported the post-acceptance manuscript processing.

## Supplementary Material

Additional File 1 List of Program Committee Members and Additional Reviewers in Alphabetical Order Click here for file

Additional File 2 A. Submission and Acceptance Statistics by Regions and Countries B. Acceptance Rate for Submissions to BMC track Click here for file
