# Supplementary material for: InCoB celebrates its tenth anniversary as first joint conference with ISCB-Asia
Source: BMC Genomics. 2011 Nov 30;12(Suppl 3):S1. doi: 10.1186/1471-2164-12-S3-S1 (PMC3333168; doi:10.1186/1471-2164-12-S3-S1)
Supplement: Additional File 1 — List of Program Committee Members and Additional Reviewers in Alphabetical Order [file 1471-2164-12-S3-S1-S1.pdf]

Additional File 1.

List of Program Committee Members and Additional Reviewers in Alphabetical Order

As editors of this conference supplement issue we wish to express our appreciation to the 76 Program Committee members and 39 additional reviewers whose expertise, timely and constructive crititiques were essential to the peer review process.

InCoB/ISCB-Asia 2011 Program Committee Members

Shandar Ahmad (National Institute of Biomedical Innovation, Japan), Tatsuya Akutsu (Kyoto University, Japan), Vladimir Bajic (King Abdullah University of Science and Technology, Saudi Arabia), Christopher J.O. Baker (University of New Brunswick, Canada) , Daniel Berrar (University of Ulster, UK) Vladimir Brusic, Dana-Farber Cancer Institute, Harvard University, USA), Filippo Castiglione (National Research Council, Italy), Alan Christoffels (South African National Bioinformatics Institute, South Africa), Anne S. De Groot (Institute of Immunology and Informatics, University of Rhode Island, and EpiVax, Inc., USA), Frank Eisenhaber (Bioinformatics Institute, A*STAR, Singapore), Andrew French (University of Nottingham, UK), Ge Gao (Center for Bioinformatics, Peking University, P.R. China) Susumu Goto (Kyoto University, Japan), M. Michael Gromiha (Computational Biology Research Center, AIST, Japan) Marsia Gustiananda (Eijkman Institute for Molecular Biology, Indonesia), Yongqun "Oliver" He (University of Michigan Medical School, USA), Chia-Lang Hsu, (National Yang-Ming University, Taiwan), Wen-Lian Hsu (Academia Sinica, Taiwan), Chun-Hsi Huang, University of Connecticut, USA), Ming-Jing Hwang (Academia Sinica, Taiwan), M Asif Khan (Johns Hopkins University, USA), Javed Khan (Macquarie University, Australia), Akira Kinjo (Osaka University, Japan), Kengo Kinoshita (Tohoku University, Japan), Akihiko Konagaya (Tokyo Institute of Technology, Japan), Gaurav Kumar (Macquarie University, Australia), Hiroyuki Kurata (Kyushu Institute of Technology, Japan), Igor V. Kurochkin (Bioinformatics Institute, A*STAR, Singapore), Vladimir Kuznetsov (Bioinformatics Institute, A*STAR, Singapore), Chee Keong Kwoh (Nanyang Technological University, Singapore), Chih Lee (University of Connecticut, USA), Ole Lund (Technical University of Denmark, Denmark), Hiroshi Mamitsuka (Kyoto University, Japan), Bill M Martin (EpiVax, Inc., USA), Hideo Matsuda (Osaka University, Japan), Amir Feisal Merican (Malaysia Genome Institute, Malaysia), Lenny Moise (EpiVax, Inc., USA), Santo Motta (University of Catania, Italy), Kenta Nakai (University of Tokyo, Japan), Haruki Nakamura (Osaka University, Japan), Sheila Nathan, (Malaysia Genome Institute, Malaysia), See-Kiong Ng (Institute for Infocomm Research, A*STAR, Singapore), Takenao Ohkawa (Kobe University, Japan), Ashwini Patil (University of Tokyo, Japan), Nikolai Petrovsky (Flinders Medical Centre, Australia), Shoba Ranganathan (PC Co-Chair; Macquarie University, Australia), Meena Sakharkar (University of Tsukuba, Japan), Daniele Santoni (Sapienza University, Italy), Christian Schönbach (PC Co-Chair; Kyushu Institute of Technology, Japan), Shahir Shamsir (Universiti Teknologi Malaysia, Malaysia), Md. Tabrez Anwar Shamim, Centre for DNA Fingerprinting and Diagnostics, India), Tetsuo Shibuya (University of Tokyo, Japan), Kazuyuki Shimizu (Kyushu Institute of Technology, Japan), Worachart Sirawaraporn (Mahidol University, Thailand), Daron M. Standley (Osaka University, Japan), Durai

Sundar (Indian Institute of Technology Dehli, India), Yoshihiro Taguchi (Chuo University, Japan), Takao Takai-Igarashi (Tokyo Medical and Dental University, Japan), Yoichi Takenaka (Osaka University, Japan), Tin Wee Tan (National University of Singapore, Singapore), Todd Taylor (RIKEN Quantitative Biology Center, Japan), Paolo Tieri (Unviversity of Bologna, Italy), Joo Chuan Tong (Institute for Infocomm Research, A*STAR, Singapore), Chandra Verma (Bioinformatics Institute, A*STAR, Singapore), Hiroshi Wako (Waseda University, Japan), Limsoon Wong (National University of Singapore, Singapore), Masayuki Yamamura (Tokyo Institute of Technology, Japan), Ueng-Chang Yang (National Yang-Ming University, Taiwan), Kei Yura (Ochanomizu University, Japan), Chao Xie (National University of Singapore, Singapore) and Guanglan Zhang (Dana-Farber Cancer Institute, Harvard University, USA).

InCoB/ISCB-Asia 2011 Additional Reviewers

Arsen Batagov (Bioinformatics Institute, Singapore), Natalia Becker (DKFZ German Cancer Research Center, Germany), Jonas Bergman Laurila (National Food Administration, Sweden), Camilo E. Lopez Carrascal (Universidad Nacional de Colombia, Colombia), Elsa Chacko (Macquarie University, Australia), Yeesiew Choong (Universiti Sains Malaysia, Malaysia), Pierre Courty (University of Basel, Switzerland), Gloria Fuentes (Bioinformatics Institute, Singapore), Jitendra Gaikwad (Macquarie University, Australia), Gagan Garg (Macquarie University, Australia), Jack Gilbert (University of Chicago, USA), Stephanus D. Handoko (Nanyang Technological University, Singapore), Frank Hauser (University of Copenhagen, Denmark), Bart Hazes (University of Alberta, Canada), Matthew Hindle (University of New Brunswick, Canada), Divya Jain (Nanyang Technological University, Singapore), Ma Jianmin (Bioinformatics Institute, Singapore), Kota Kasahara (Tohoku University, Japan), Dimitar Kenanov (Bioinformatics Institute, Singapore), Varun Khanna (Macquarie University, Australia), Shinji Kondo (RIKEN Omics Science Center, Japan), Lesheng Kong (University of Oxford, UK), Dan Larhammar (Uppsala University, Sweden), Vachiranee Limviphuvadh (Bioinformatics Institute, Singapore), Janez Mavri (National Institute of Chemistry, Slovenia), Saharuddin Mohamad (University of Malaya, Malaysia), Efthimios Motakis (Bioinformatics Institute, Singapore), Shivashankar Nagaraj (CSIRO Livestock Industries, Australia), Baskaran Natesan (National Institute of Technology Tiruchirappalli, India), Thuy D. Nguyen (Nanyang Technological University, Singapore), Lars Olsen (University of Copenhagen, Denmark), Philip Prathipati (Bioinformatics Institute, Singapore), Masakazu Sekijima (Computational Biology Research Center, AIST, Japan), Westley A. Sherman (Bioinformatics Institute, Singapore), Matsuyuki Shirota (Tohoku University, Japan), Nicolas Soler (Institut Cochin, France), Zhiqun Tang (Bioinformatics Institute, Singapore), Ikuo Uchiyama (National Institute for Basic Biology, Japan) and Rui Yamaguchi (University of Tokyo, Japan).
