# Supplementary material for: InCoB celebrates its tenth anniversary as first joint conference with ISCB-Asia
Source: BMC Genomics. 2011 Nov 30;12(Suppl 3):S1. doi: 10.1186/1471-2164-12-S3-S1 (PMC3333168; doi:10.1186/1471-2164-12-S3-S1)
Supplement: Additional File 2 — A. Submission and Acceptance Statistics by Regions and Countries B. Acceptance Rate for Submissions to BMC track [file 1471-2164-12-S3-S1-S2.pdf]

Additional file 2

A. Submission and Acceptance Statistics by Regions and Countries

Region/Country	Submissions BMC	IR	BI	Total	Accepted BMC	IR	BI	Total
East Asia	34 (32.7)	1	1	36	20 (40.8)	1	1	22
Japan	9 (8.6)	-	1		6 (12.2)	-	1	
RO Korea	5 (4.8)	-	-		4 (8.1)	-	-	
Taiwan	14 (13.5)	-	-		7 (14.3)	-	-	
PR China	6 (5.8)	1	-		3 (6.1)	1	-	
South Asia	25 (24.0)	-	1	26	6 (5.8)	1	2	9
Bangladesh	1 (1.0)	-	-		0 (0.0)	-	1*	
India	22 (21.2)	-	1		6 (5.8)	1*	1	
Sri Lanka	2 (1.9)	-	-		0 (0.0)	-	-	
Southeast Asia	27 (26.0)	1	1	29	14 (28.6)	1	1	16
Indonesia	1 (1.0)	1	-		0 (0.0)	1	-	
Malaysia	11 (10.6)	-	1		4 (8.2)	0	1	
Singapore	12 (11.5)	-	-		10 (20.4)	-	-	
Thailand	2 (1.9)	-	-		0 (0.0)	-	-	
Vietnam	1 (1.0)	-	-		0 (0.0)	-	-	
Europe	4 (3.8)	-	-	4	3 (6.1)	-	-	3
Germany	1 (1.0)	-	-		1 (2.0)	-	-	
Italy	1 (1.0)	-	-		1 (2.0)	-	-	
UK	2 (1.9)	-	-		1 (2.0)	-	-	
North America	7 (6.7)	1	-	8	3 (6.1)	1	1*	5
Canada	1 (1.0)	-	-		0 (0.0)	-	-	
USA	6 (5.8)	1	-		3 (6.1)	1	1*	
Oceania	6 (5.8)	-	-	6	3 (6.1)	-	-	3
Australia	6 (5.8)	-	-		3 (6.1)	0	-	
South America	1 (1.0)	-	-	1	0 (0.0)	-	-	0
Brazil	1 (1.0)	-	-		0 (0.0)	-	-	
	104 (100.0)	3	3	110	49 (100.0)	4	5	58

BMC (BMC Genomics and BMC Bioinformatics; IR: Immunome Research; BI: Bioinformation; numbers in parenthesis indicate percentage; submissions that were transferred from BMC tracks to IR or BI are marked with an asterisk.

B. Acceptance Rate for Submissions to BMC track

	No. accepted/submitted	[%]
East Asia	20/34	58.8
Japan	6/9	66.7
RO Korea	4/5	80.0
Taiwan	7/14	50.0
PR China	3/6	50.0
South Asia	6/25	24.0
Bangladesh	0/1	0.0
India	6/25	24.0
Sri Lanka	0/2	0.0
Southeast Asia	14/27	51.9
Indonesia	0/1	0.0
Malaysia	4/11	36.4
Singapore	10/12	83.3
Thailand	0/2	0.0
Vietnam	0/1	0.0
Europe	3/4	75.0
Germany	1/1	100.0
Italy	1/1	100.0
UK	1/2	50.0
North America	3/7	42.9
Canada	0/1	0.0
USA	3/6	50.0
Oceania	3/6	50.0
Australia	3/6	50.0
South America	0/1	0.0
Brazil	0/1	0.0
Total	49/104	47.1
